# The Association Between Helicobacter Pylori and Perforated Gastroduodenal Ulcer

**DOI:** 10.7759/cureus.7406

**Published:** 2020-03-25

**Authors:** Katavath Thirupathaiah, Loganathan Jayapal, Anandhi Amaranathan, Chellappa Vijayakumar, Mangala Goneppanavar, Vishnu Prasad Nelamangala Ramakrishnaiah

**Affiliations:** 1 Surgery, Jawaharlal Institute of Postgraduate Medical Education and Research (JIPMER), Puducherry, IND; 2 Surgery, Lala Lajpat Rai Memorial Medical College, Meerut, IND; 3 Pathology, Mahatma Gandhi Medical College and Research Institute, Puducherry, IND

**Keywords:** helicobacter pylori, peptic ulcer, smoking, nsaid, alcohol

## Abstract

Background

Although the role of Helicobacter pylori (H. pylori) is well known in peptic ulcer disease (PUD) etiology, its role in perforated peptic ulcer (PPU) is not well established. This study aimed to assess the role of H. pylori infection in patients with PPU and to compare it with its prevalence in patients with PUD.

Methodology

This prospective analytical study was conducted for two years. The study patients were classified into two groups: group I consisted of patients (n = 48) operated for PPU and group II were patients (n = 48) with PUD diagnosed by endoscopy. The study excluded patients with any previous anti-H. pylori treatment, gastric malignancy, conservative management of sealed PPU, and those with a diameter of perforation less than the diameter of endoscopic biopsy forceps. Blood samples were taken for H. pylori serology IgG (ELISA) test. The mucosal biopsy samples from both the groups were tested with a rapid urease test and modified Giemsa stain examination to detect H. pylori.

Results

The prevalence of H. pylori infection were significantly less in patients with PPU than with PUD: by rapid urease: 12.5% vs. 31.2%, p: 0.002; modified Giemsa stain: 10.4% vs. 31.2%, p: 0.012; and IgG serology: 50 % vs. 68.7%, p: 0.012, respectively. Alcohol and tobacco were significant risk factors (p: 0.002 vs. p: 0.002 respectively). However, nonsteroidal anti-inflammatory drugs (NSAIDs) use was not a significant risk factor for PPU (p: 0.083).

Conclusion

H. pylori infection was not significantly associated with PPU. Some other factors like alcohol intake and tobacco were also involved in perforation. We can conclude that H. pylori infection is not a risk factor for PPU.

## Introduction

The incidence of perforated peptic ulcer (PPU) has only declined minimally despite the widespread use of Helicobacter pylori (H. pylori) eradicative agents. About 95% of the patients suffering from duodenal ulcers and 70-80% of the gastric ulcer patients are found to be H. pylori-positive [1]. Although the role of H. pylori is well known in peptic ulcer disease (PUD) etiology, its role in PPU is not well established [2-5]. There are conflicting results in the literature regarding its association. While some studies show a high prevalence of H. pylori infection in PPU patients, and its eradication preventing the relapse of ulcer, others show a low or complete lack of association, suggesting different pathogenesis for PPU [1,6-7]. Because of these conflicting results and paucity of data, this study was undertaken to assess the role of H. pylori in patients with PPU.

## Materials and methods

This study aimed to assess the role of H. pylori infection in patients with PPU and to compare it with its prevalence in patients with PUD. This was a prospective analytical study conducted at a tertiary care center in South India for 17 months. Study patients were divided into two groups. Group I included patients above 18 years of age who were operated for gastro-duodenal perforation. Group II consisted of patients above 18 years of age with the upper gastrointestinal endoscopic diagnosis of PUD (peptic ulcer or erosive mucosal disease).

In the first group, the mucosal biopsy was taken during surgery from the antrum of the stomach through the perforation using sterile endoscopic biopsy forceps. In group ll, after the endoscopic diagnosis of PUD, the mucosal biopsy was taken from the antrum of the stomach. The study excluded patients with any previous anti-H. pylori treatment, gastric malignancy, conservative management of sealed peptic perforation, and those with a diameter of perforation less than the diameter of endoscopic biopsy forceps. The biopsy samples from both the groups were subjected to rapid urease test and histopathological (modified Giemsa stain) examination to detect H. pylori. The patients were considered to have H. pylori infection if any one or both of the above tests were positive. Smokers were defined as patients who were smoking during the study and had smoked more than 100 cigarettes in the past year. Drinkers were defined as patients who had consumed alcohol in the past 12 months. Nonsteroidal anti-inflammatory drugs (NSAIDs) intake was defined as the current intake of NSAIDs in patients irrespective of the dosage.

Statistical analysis

The results of the primary outcome variables (H. pylori-positive PPU and PUD) and sociodemographic and clinical variables (age group, gender) were presented as absolute numbers and percentages. The difference between the two groups, with and without H. pylori infection, was assessed using the Chi-square test, and a p-value of <0.05 was considered statistically significant.

## Results

A total of 96 patients (48 patients in each group) was included in the study. The male-to-female ratio between the study groups was 15:1 and 3.8:1 respectively. The mean age of presentation was comparable between the groups (46.5 ±16 years vs. 47.3 ±13 years). The highest incidence of PPU was found in patients of 31-40 years age in group l and those of 51-60 years age in group II. Among them, 22 (45.8%) patients were smokers, 27 (56.3%) patients had a history of alcohol consumption, and 26 (54.2%) patients had a history of NSAIDs use (Table 1).

**Table 1:** Baseline parameters of the study groups NSAIDs: nonsteroidal anti-inflammatory drugs; SD: standard deviation

Parameter	Group I (n = 48)	Group II (n = 48)	P-value
Age in years, mean ±SD	46.5 ±2	47.3 ±2	-
Sex ratio (male-to-female)	15:1	3.8:1	-
Smoking, n (%)	22 (45.8)	8 (16.7)	0.002
Alcohol intake, n (%)	27 (56.3)	12 (25)	0.002
NSAIDs intake, n (%)	26 (54.2)	24 (50)	0.68

A significant statistical difference existed between group I and group II when alcohol intake and smoking were considered as positive risk factors (p: 0.002), as laid out in Table 2.

**Table 2:** Distribution of risk factors between the study groups NSAIDs: nonsteroidal anti-inflammatory drugs

Risk factors	Group l (%)	Group ll (%)
Alcoholic intake	56.25	14.3
Smoking	45.83	16.67
NSAIDs intake	54.17	50

Out of the 48 patients in group I, 42 (87.5 %) patients had pre-pyloric perforation, and six patients (12.5%) had a perforation in the first part of the duodenum. Out of the 48 patients in group ll, 14 patients had antral gastritis, 14 patients had duodenitis, 17 patients had pan gastritis, and three patients had pre-pyloric ulcers (Table 3).

**Table 3:** Relationship between upper gastrointestinal endoscopy findings and H. pylori status in patients of peptic ulcer or erosive mucosal disease (group II) H. pylori: Helicobacter pylori

Diagnosis	H. pylori-positive (n)	H. pylori-negative (n)
Pre-pyloric ulcer	1	2
Duodenitis	6	8
Antral gastritis	8	6
Pangastritis	1	16

H. pylori was detected in all three tests in 15 (31.3%) patients. Only serology was positive in 18 patients. The prevalence of H. pylori was significantly less [p: 0.026; odds ratio (OR): 0.31] in PPU patients compared to PUD or mucosal erosive disease patients (Table 4).

**Table 4:** Significance of H. pylori in group I and group II based on investigations for the detection of H. pylori H. pylori: Helicobacter pylori

Investigations			Group I (n = 48)	Group II (n = 48)	Total	P-value
Rapid urease, n (%)	H. pylori	Positive	6 (12.5)	15 (31.25)	21 (21.87)	0.026
		Negative	42 (87.5)	33 (68.75)	75 (78.12)	
Modified Giemsa stain, n (%)	H. pylori	Positive	5 (10.41)	15 (31.25)	20 (20.83)	0.012
		Negative	43 (89.58)	33 (68.75)	76 (79.16)	
Serology IgG, n (%)	H. pylori	Positive	24 (50)	33 (68.75)	57 (59.37)	0.061
		Negative	24 (50)	15 (31.25)	39 (40.62)	

## Discussion

Although the chronicity and recurrence of peptic ulcers are strongly associated with H. pylori infection as shown in many studies, does it mean that the infection should be associated with PPU also? The present study was carried out to study the association between H. pylori infection and PPU. Males were found to be predominantly affected with a male to female ratio of 15:1. This was similar to a study done by Ugochukwu et al. and Dogra et al. where males were found to be more commonly affected with a ratio of 3.2:1 and 3:1 respectively [8,9]. Also, the mean age of presentation in both these studies was 39.5 ±13 years and 49.2 years respectively. In another study done by John et al., the mean age was 52.81 ±14 years with a male to female ratio of 4.14:1, which is similar to the present study [10].

The present study shows that the prevalence of H. pylori infection among patients presenting with a PPU was 12.5%. Among them, 87.5% had a perforation in the pre-pyloric region and 12.5% were in the first part of the duodenum. The prevalence of H. pylori infection among patients with PUD was 31.3%. This was based on the rapid urease test and histopathological examination. The results were statistically significant with a p-value of 0.026 when H. pylori was considered as an exposure factor, and with an odds ratio (OR) of 0.31; we found that H. pylori infection was not significantly associated with PPU. This is similar to the study done by Gisbert et al. in which the prevalence of H. pylori infection in PPU was significantly less with an infection rate of 47% [11]. They found that chronic recurrent PUD and PPU have different pathogenesis based on the fact that there is a low prevalence of H. pylori infection in patients presenting with PPU. It also suggests that other pathogenic factors might also play a role in PPU [12]. Also, another study done by Gisbert et al. found that all their 15 patients with PPU were negative for H. pylori [13]. But in contrast to our study, most other studies showed a significant association of H. pylori infection with PPU. The discrepancy in the infection rates found in the literature may be attributed in part to the different populations studied.

In the present study, 45.8% of patients in group I were smokers, among which 13.63% tested positive for H. pylori. In group II, only 16.66% were smokers of which only one patient was tested positive for H. pylori infection. Alcohol intake is another attributable risk factor for PPU. In the present study, 56.3% of PPU patients had a history of alcohol intake and among them, two were tested positive for H. pylori. In PUD patients, 25% had a history of alcohol consumption of which five were found to be positive for H. pylori. The results were statistically significant (p: 0.002) when alcohol intake and smoking were considered as risk factors for PPU. Based on the OR (3.85 and 4.23, respectively), it was found that both alcohol intake and smoking were risk factors for PPU. This was similar to a study done by Ugochukwu et al., which showed that smoking and alcohol consumption were significantly associated with perforation in young men from developing countries [8]. Previous studies that show a significant association between H. pylori infection and PPU are listed below (Table 5) [8-10,12].

**Table 5:** Previous studies showing significant association between H. pylori infection and PPU H. pylori: Helicobacter pylori; PPU: perforated peptic ulcer

Study	Year	Prevalence of H. pylori infection in PPU, %	Association
Ugochukwu et al. [8]	2013	65-70	Significant
Dogra et al. [9]	2014	92	Significant
John B et al. [10]	2017	47	Significant
Sebastian et al. [12]	2001	83.3	Significant

## Conclusions

We found that alcohol intake and smoking were significant risk factors associated with perforation of gastro-duodenal ulcers, whereas NSAIDs use was not significantly associated. H. pylori infection was not significantly associated with PPU, implying that some other factors were also involved in perforation.
